# Comparison of compliance among patients with pediatric amblyopia undergoing virtual reality-based and traditional patching method training

**DOI:** 10.3389/fpubh.2022.1037412

**Published:** 2022-10-14

**Authors:** Li Li, Hailan Xue, Taichen Lai, Ying Xue, Gang Luo

**Affiliations:** ^1^Ophthalmology Department, Fujian Provincial Hospital South Branch, Fujian Provincial Hospital, Fuzhou, China; ^2^Schepens Eye Research Institute, Harvard Medical School, Boston, MA, United States; ^3^Shengli Clinical Medical College of Fujian Medical University, Fuzhou, China; ^4^Ultrasound Department, Fujian Maternity and Child Health Hospital, College of Clinical Medicine for Obstetrics & Gynecology and Pediatrics, Fujian Medical University, Fuzhou, China

**Keywords:** amblyopia, amblyopia training, compliance, EYEBIT, virtual reality

## Abstract

**Introduction:**

This study aimed to compare compliance between pediatric patients with amblyopia undergoing a smartphone virtual reality-based training method (EYEBIT) and those receiving traditional patching method training.

**Methods:**

A crossover design was adopted in this study. The enrolled children (*n* = 76) were randomized into the traditional patching and EYEBIT training method groups. The patients received training methods once a day for 2 h and 1 h in the patching and EYEBIT groups, respectively. Follow-up assessments involved interviews with parents regarding children's compliance and questionnaire-based interviews with children; compliance rating was compared between the methods.

**Results:**

All children completed the training and follow-up assessments. There were significant differences in parent and children compliance-related behavior and attitudes between the two training methods (*p* < 0.05). The EYEBIT method was associated with better compliance than the traditional patching method. Significant correlations were observed among compliance components in both methods. In the correlation analysis between the two groups, the research results showed that in the EYEBIT group, the correlation between children's compliance behaviors and children's compliance attitudes, the correlation between children's compliance behaviors and parents' compliance behaviors, and the correlations between children's compliance attitudes and parents' compliance attitudes were all negatively correlated, and in the traditional patching group, the above three correlation analysis results were all positive.

**Conclusion:**

The use of the EYEBIT method may improve compliance in children with amblyopia; this method appears acceptable to the parents of children with amblyopia.

## Introduction

Amblyopia is a common eye disease in children associated with impairments in processing tactile, motion, and visual stimuli, resulting in abnormal visual development, low visual acuity, and binocular diplopia (1–3). Amblyopia was once considered a monocular disease; however, a growing body of evidence suggests the involvement of structural and functional abnormalities in both eyes (4). Pediatric amblyopia is associated with high financial and psychological burden to both children and parents.

Treatment choices depend on children's age, visual acuity, compliance, and response to previous treatment, as well as children's physical, social, and psychological development. Treatment methods include refractive correction, traditional patching, atropine fogging, filter, optical fogging, and dichoptic training (5–8). The patching method remains the most popular.

A growing body of research has exposed the limitations of these treatment methods (9). Traditionally, amblyopia has been attributed to habitual monocular suppression and irreversible decrease in cortical excitability of bilateral visual V1 neurons (10); however, current research suggests the involvement of other mechanisms. For example, Sengpiel et al. (11) found that in the animal model of strabismus, the suppression of binocular visual acuity recovered after using GABA antagonist; meanwhile, the function loss of V1 neurons in binocular visual acuity response was reversible. Cotter et al. (12) also found in clinical experiments that if only the contrast of the eye with better visual acuity in patients with amblyopia was reduced, the patients retained binocular visual acuity through the complete binocular visual cortex mechanism, while active monocular suppression led to functional binocular suppression despite intact visual system structures. These results indicate that the loss of binocular visual function may result from active suppression, suggesting that dichoptic training may support the recovery of binocular visual function.

Mezad-Koursh et al. (13) used the video import system to conduct eye patching dichoptic training, which improved the visual acuity of children with amblyopia. Birch et al. (14) and Li et al. (15) used iPad for dichoptic training to treat amblyopia using games, aiming to improve the rates of treatment compliance among children. The evidence presented by these authors suggests that this approach may improve short-term visual acuity with efficacy greater than that of the traditional patching method. Kelly et al. (16) found that in children with amblyopia treated by dichoptic training for 2 weeks, both suppression depth and degree had improved, with greater effects observed in children with poorer suppression depth.

Based on previous research, we adopted the EYEBIT mobile phone software system developed by Gang at the Schepens Eye Research Institute of Harvard Medical School, combined with virtual reality technology, for training patients with pediatric amblyopia. This approach was used to eliminate the shortcomings of approaches previously used in China and elsewhere for dichoptic training to treat amblyopia. To support future studies, this study aimed to evaluate compliance rates associated with this training system.

## Materials and methods

### EYEBIT modules

EYEBIT consists of five modules.

Video playback module: (1) Play local video; (2) Switch videos in virtual reality environment; (3) To realize the simultaneous training method for amblyopia treatment, different sizes of central covering areas can be set in both eyes; (4) In the virtual reality environment, the two windows can move in the same direction or in the opposite direction to achieve the function of training fusion.

Screen sharing module: (1) To realize the screen sharing function between mobile phones, parents can observe the image situation of children's training through the shared mobile phone; (2) Research physicians or family members can control the images and training time of children through sharing mobile phones.

Parameter control module: (1) Unified management of all user parameters, can achieve global access; (2) It can read and write files in a fixed format to realize the function of reading the parameters of the treatment plan sent remotely by doctors; (3) parameter format control.

Head movement detection module: it can recognize simple head movement posture, such as turning head left, turning head right, raising head, and lowering head, to flexibly control the picture and switch the video during training.

Server management module: (1) Upload valid user training duration; (2) Upload masks with different degrees of ambiguity used by users in different times of training.

### EYEBIT training

**Figure 1:**
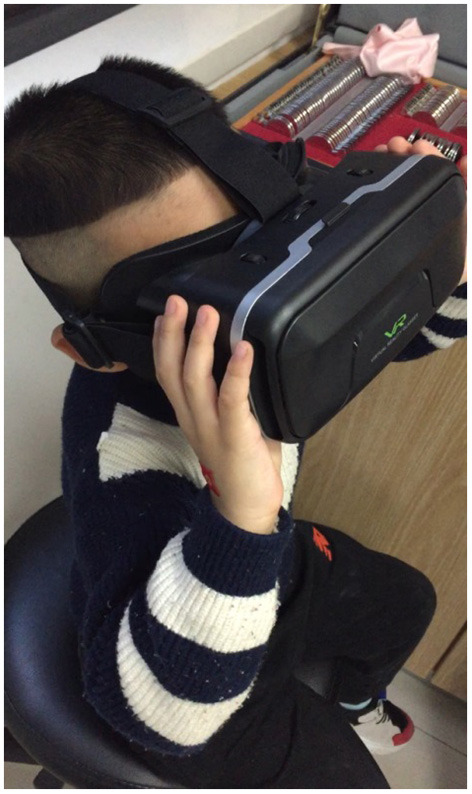
Training scene of the EYEBIT system based on virtual-reality (VR) technology.

**Figure 2:**
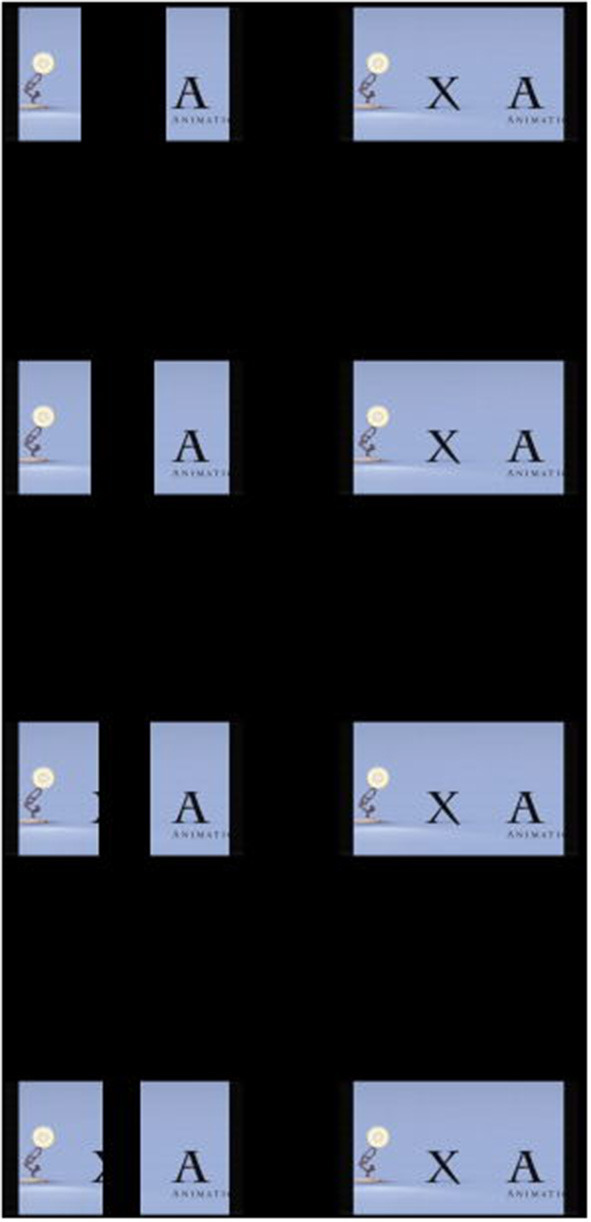
Comparison chart after adding four masks of different degrees to the left eye window.

EYEBIT is a mobile app that plays a video stream in two windows on the phone screen to allow the video to be watched through a pair of virtual reality goggles (Figure 1). For amblyopia therapy, the window corresponding to the good eye is partially covered by the central area, while the other window for the amblyopic eye retains the original contrast. Before training, the patient saw two identical pictures in both the left and right windows of the virtual reality glasses case. The innovative use of partial covering of the central area of the dominant eye window enables the two eyes to get the complete picture by increasing the visual training and stimulation of the central vision of the amblyopia eye (Figure 2).

**Figure 3:**
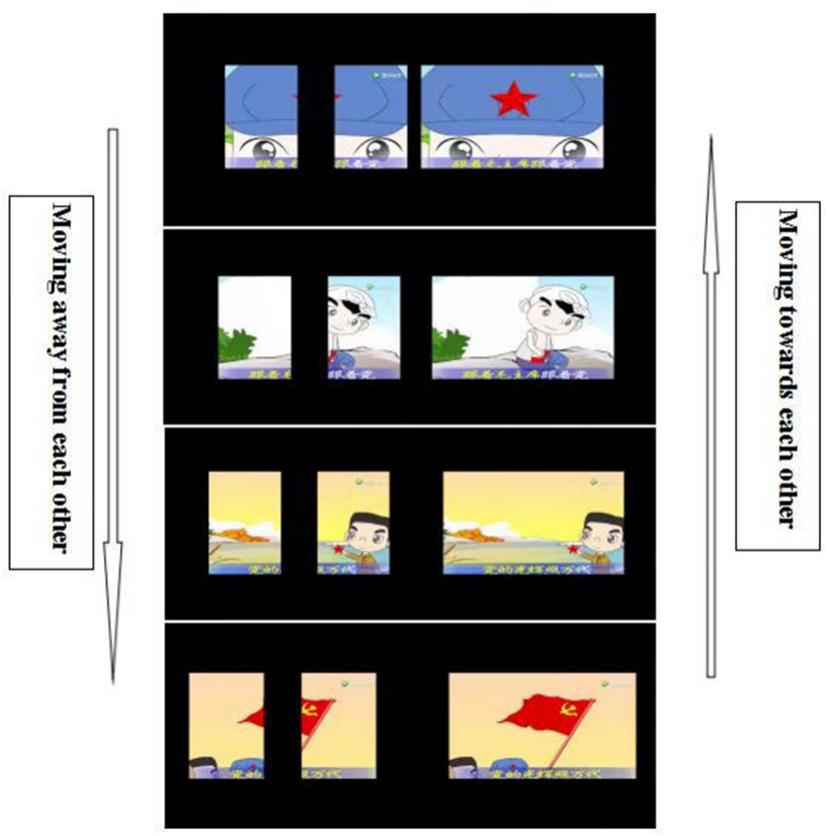
The two viewing windows move toward and away from each other to create changing vergence demands during vision training.

Most children with amblyopia have reduced binocular fusion function; thus, the EYEBIT system was designed to shift the two viewing windows toward and away from each other. The amount and frequency of shift were set according to patients' fusion function. The shift was specified in prism diopter (typically 2 PD) and the frequency of shift in times/minutes (typically 20/min). Thus, during the amblyopia training, the patients also underwent fusion training (Figure 3), which may help improve their binocular visual function.

Using EYEBIT, patients were able to select the videos for their training according to their personal preferences. The viewing distance, magnification, fusion change step, and interpupillary distance were set by clinicians.

### Eligibility criteria

The study was approved by the hospital ethics committee (K2018-12-031). Children aged 4–14 years who were diagnosed by an ophthalmologist as having anisometropic amblyopia, refractive amblyopia, deprivation amblyopia, and strabismic amblyopia were screened. Visual acuity was examined according to the international standard visual acuity chart; the best-corrected visual acuity of the amblyopic eye was 0.1–0.5, and that of the dominant eye was higher than or equal to 0.8 (best-corrected visual acuity of the dominant eye of children under 5 years was higher than or equal to 0.6); the difference in best-corrected visual acuity values between the two eyes was more than two lines in the standard logarithmic visual acuity chart. For strabismic amblyopia, the degree of strabismus was ≤ 5 PD after optical correction or surgical treatment. Children were eligible for this study provided that their parents were willing to consent to study participation and cooperate with study activities, including follow-up assessments.

Among children with amblyopia, those with major systemic hereditary diseases (e.g., congenital heart disease, congenital growth retardation) were excluded; those with other ocular organic diseases, e.g., congenital cataract, congenital glaucoma, and corneal diseases, were excluded; children with a history of atropine treatment for amblyopia, gestational age at birth of <32 weeks, or birth weight of <1.5 kg were excluded.

### Compliance assessment

All patients were instructed to take 2-h traditional patching therapy and also 1-h EYEBIT training every day. The order of the two training methods was fully randomized. For one subject, the order may or may not change the next day. Overall, each training method had an ~50% chance to be used prior to the other across the entire study duration. The patients' parents were instructed to supervise the therapy and implemented the training order according to random order sheets assigned to their children. The compliance of both children and parents was investigated. During the 1-month follow-up, the parents and children were administered questionnaires to report compliance of the children and parents themselves. In addition, parents' and children's perception and psychological acceptance of the two training methods were also surveyed.

The questionnaires are provided in the Appendix. In summary, the parents reported their children's compliance with each training method on the first day, in the first week, and the first month overall. The parents reported their compliance to their supervision duty for each training method in terms of completion of training and regularity of schedule. However, due to the influence of games or videos, most children are easily attracted by the EYEBITt method on the first day and the first week. Some children do not directly cooperate in the traditional patching method, and the error of compliance analysis results has a large impact. For these patients, we suggest that they can watch the same games or videos during the covering method training, and record and analyze the compliance in the first month after the stable training. Compliance was rated on a 5-point scale: strongly disagree, disagree, neutral, agree and strongly agree. Score: 1 point for strongly disagree, 2 points for disagree, 3 points for neutral, 4 points for agree, and 5 points for strongly agree.

Both parents and children groups reported their psychological acceptance, as well as their perception of the helpfulness, of each training method. In addition, parents also reported their preference for the two methods. Responses were rated on a 5-point scale.

### Statistical analysis

The reliability and validity of the compliance questionnaire were examined. The validity of a tool represents its suitability for a particular study. The reliability of a tool represents its ability to provide consistent results. An independent sample *t*-test was used to compare compliance scores between the training methods. Finally, the Pearson product-moment correlation analysis was used to examine associations among compliance components. The Pearson correlation coefficient was used to examine associations among variables.

## Results

Between October 1, 2018, and January 18, 2021, this study included 76 (41 females) patients with an average age of 8.1 years. According to the diagnostic criteria for amblyopia provided by the Chinese Association for Pediatric Ophthalmology and Strabismus at the Chinese Ophthalmological Society, there were 58, 3, and 15 cases of anisometropic amblyopia, strabismic amblyopia, and ametropic amblyopia, respectively. And record other information of the patient (Table 1).

**Table 1:** Patient information.

**Patient information**		**Number of cases**
Gender	Female	41
	Male	35
Residence	Urban	57
	Rural	19
Parent's degree	High school education	8
	Undergraduate education	51
	Graduate education	17
Type of amblyopia	Anisometropic amblyopia	58
	Strabismic amblyopia	3
	Ametropic amblyopia	15
Premature infant	Yes	10
	No	66
Family history of amblyopia	Yes	7
	No	69

### Questionnaire validity

The Kaiser–Meyer–Olkin (KMO) values for the traditional patching method and EYEBIT method were 0.766 and 0.723, respectively, suggesting a strong partial correlation and indicating that the scale was suitable for factor analysis (Table 2). Bartlett's sphericity test result had a *p*-value of < 0.001, suggesting a correlation between variables, which supports the suitability of the scale for factor analysis and suggests that it has good validity.

**Table 2:** KMO and Bartlett's test.

**Training method**		**Traditional patching method**	**EYEBIT method**
KMO measure of sampling adequacy		0.766	0.723
Bartlett's test of sphericity	Approximate chi-square	388.909	301.626
*df*		55	55
Sig.		0.000	1.0

KMO, Kaiser–Meyer–Olkin.

### Reliability test

Cronbach's alpha values for compliance components in the traditional patching and EYEBIT methods were 0.854 and 0.850, respectively, indicating satisfactory internal consistency and data accuracy (Table 3).

**Table 3:** Reliability test results.

**Dimension**	**Cronbach's alpha**	**Number of terms**
Patching	0.854	11
EB	0.850	11

EB, EYEBIT.

### Differences in compliance between the two training methods

**Figure 4:**
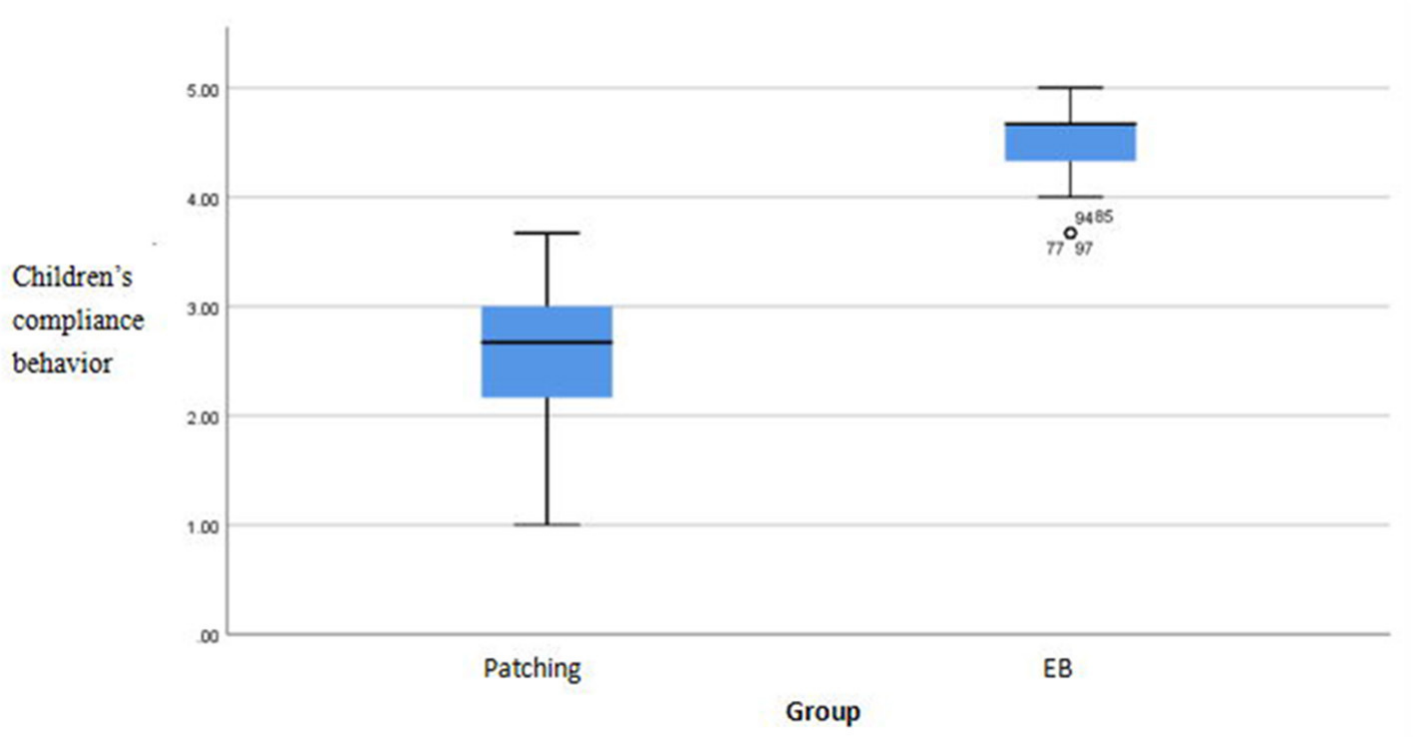
Box plots of children's compliance behavior on two training methods. EB, EYEBIT.

**Figure 5:**
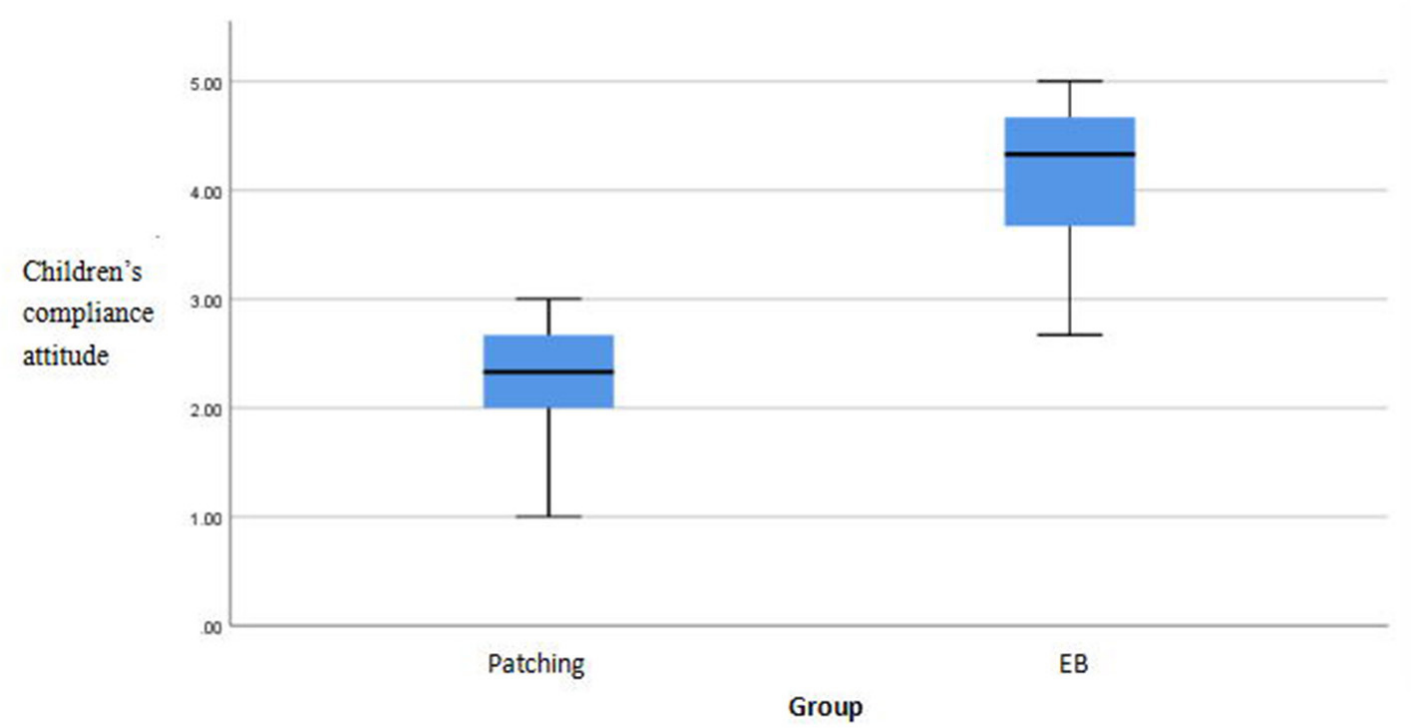
Box plots of children's compliance attitude on two training methods. EB, EYEBIT.

**Figure 6:**
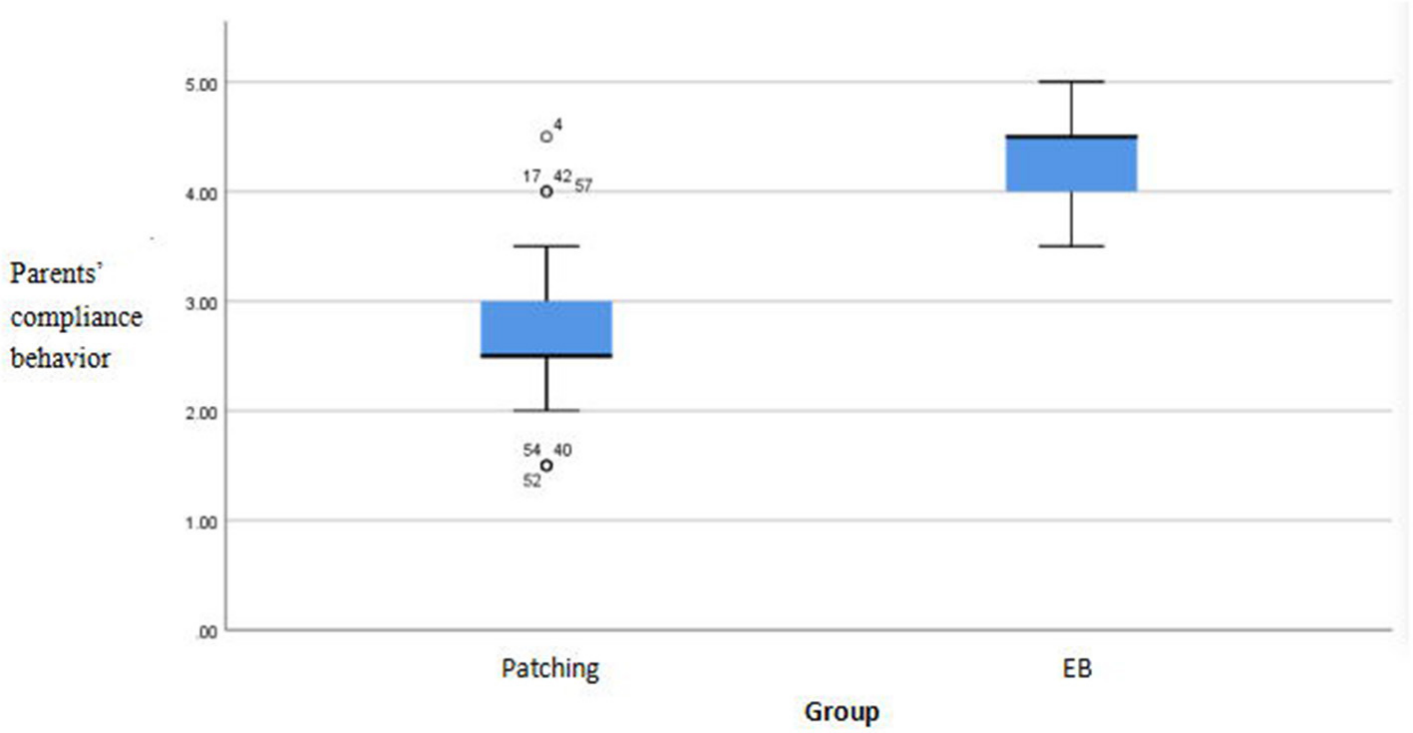
Box plots of parents' compliance behavior on two training methods. EB, EYEBIT.

**Figure 7:**
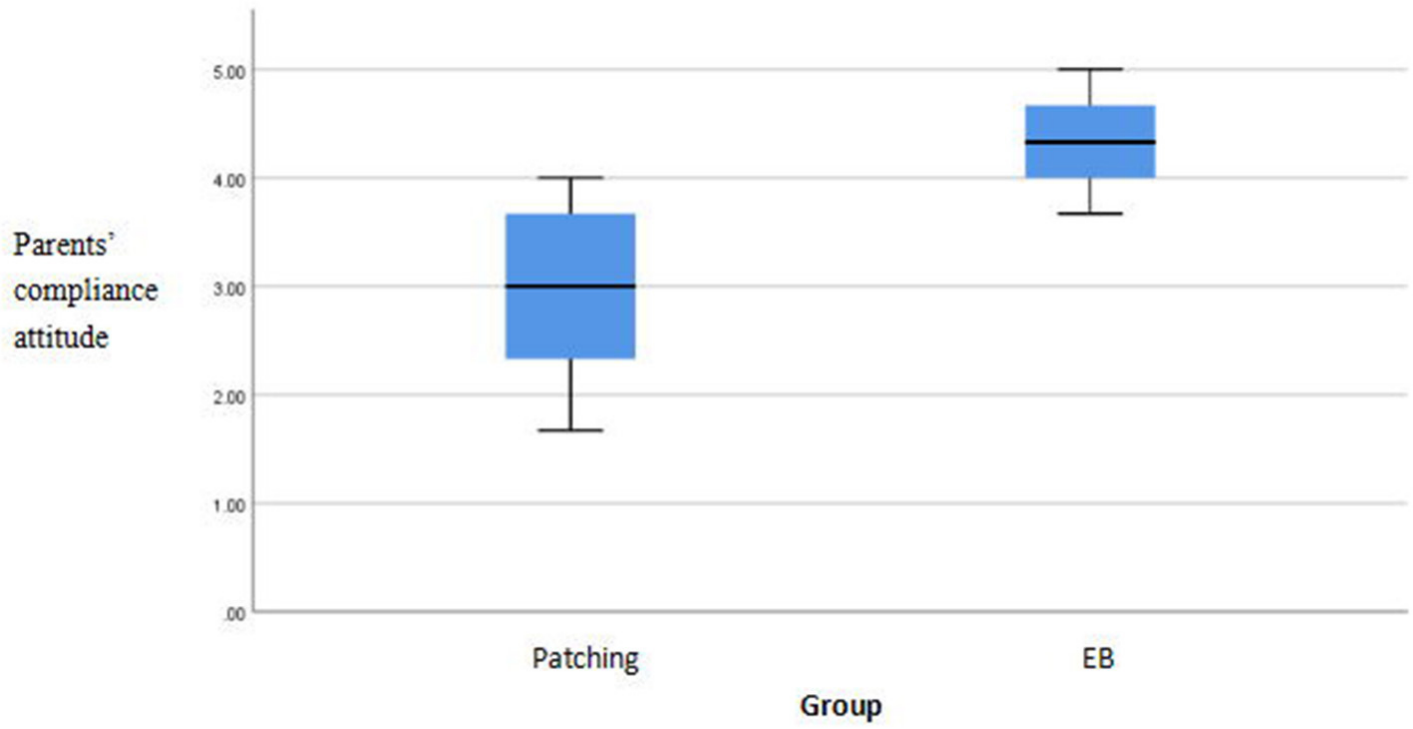
Box plots of parents' compliance attitude on two training methods. EB, EYEBIT.

There were significant differences in compliance scores between the methods (*p* < 0.05). The EYEBIT method was associated with better compliance than the traditional patching method (Table 4). Both methods are shown in the box plot, and the scores of the EYEBIT method in compliance behavior and attitude of parents and children are higher than those of the traditional patching method (Figures 4–7).

**Table 4:** Compliance score differences between the two training methods.

**Compliance**	**Group**	**Mean**	**SD**	***t***	**Sig. (two-sided)**
Children's compliance behavior	Patching	2.43	0.69	−21.939	0.000
	EB	4.46	0.41		
Children's compliance attitude	Patching	2.27	0.47	−19.767	0.000
	EB	4.07	0.64		
Parents' compliance behavior	Patching	2.68	0.64	−18.644	0.000
	EB	4.35	0.44		
Parents' compliance attitude	Patching	2.88	0.69	−15.990	0.000
	EB	4.36	0.42		

EB, EYEBIT.

### Correlations among compliance components

The Pearson correlation coefficient between children's compliance behavior, children's compliance attitude, and parents' compliance behavior was 0.405 and 0.234, respectively, under the traditional patching method. The Pearson correlation coefficient between parents' compliance attitude, children's compliance attitude, and parents' compliance behavior was 0.285 and 0.302, respectively. The Pearson correlation coefficient between the compliance behavior of children under the EYEBIT method and the compliance behavior of children under the traditional patching method was 0.252. The Pearson correlation coefficients between compliance attitude of children with the EYEBIT method and compliance behavior and attitude of children with the traditional patching method and compliance behavior and attitude of parents were −0.449, −0.332 and −0.375, −0.261, respectively. The Pearson correlation coefficient between parental compliance attitude under the EYEBIT method and parental compliance attitude under the traditional patching method was 0.479, and that between parental compliance attitude and child compliance behavior under the EYEBIT method was 0.328. The EYEBIT method has a significant and moderate positive correlation with the compliance attitude of parents under the traditional patching method, showing a tendency of consistency in the compliance attitude of parents. However, there was a significant negative correlation between the compliance attitude of children with the EYEBIT method and the compliance behavior and attitude of children with the traditional patching method, as well as the compliance behavior and attitude of parents (Table 5).

**Table 5:** Correlation analysis of different compliance under the EYEBIT method and the traditional patching method.

	**Children's compliance behavior (patching)**	**Children's compliance attitude (patching)**	**Parents' compliance behavior (patching)**	**Parents' compliance attitude (patching)**	**Children's compliance behavior (EB)**	**Children's compliance attitude (EB)**	**Parents' compliance behavior (EB)**	**Parents' compliance attitude (EB)**
Children's compliance behavior (patching)	1							
Children's compliance attitude (patching)	0.405^**^	1						
Parents' compliance behavior (patching)	0.234^*^	0.067	1					
Parents' compliance attitude (patching)	0.213	0.285^*^	0.302^**^	1				
Children's compliance behavior (EB)	0.252^*^	0.19	0.165	0.142	1			
Children's compliance attitude (EB)	−0.449^**^	−0.332^**^	−0.375^**^	−0.261^*^	−0.147	1		
Parents' compliance behavior (EB)	−0.125	0.019	0.124	−0.069	−0.141	0.207	1	
Parents' compliance attitude (EB)	0.122	0.097	0.193	0.479^**^	0.328^**^	−0.034	0.018	1

** Significantly correlated at the level of 0.01 (two-sided).

* Significantly correlated at the level of 0.05 (two-sided). EB, EYEBIT.

## Discussion

Amblyopia is the most common cause of monocular visual impairment in children, with a prevalence rate of 2%−3% (17). Affected patients experience reduced visual acuity in one or both eyes. The disease also reduces eye-hand coordination and reading ability, negatively affecting the quality of life and self-esteem of patients (17). Traditional amblyopia treatment methods have many disadvantages. On the one hand, the traditional patching method, which involves patching the good eye and forcing the amblyopic eye to receive training, has been associated with poor compliance in children with extensive binocular visual acuity difference or relatively poor visual acuity of the amblyopic eye. On the other hand, excessive suppression of children's dominant eye may also lead to the reversal of visual acuity of strong and weak eyes, resulting in occlusion amblyopia, hindering the recovery of binocular visual function. Atropine can be used to blur near vision and force the amblyopic eye to receive near vision training. However, this approach does not affect distant vision, and atropine may cause side effects, such as cysts and nodules, in the eyes.

To improve treatment appeal, compliance, and outcomes, research efforts have focused on developing novel training methods, including iPad-based games proposed by Li et al. (9). This dichoptic training method mainly treats amblyopia by weakening the contrast of the dominant eye, thus allowing dichoptic training after binocular contrast rebalancing. This binocular contrast rebalancing helps improve binocular suppression and binocular vision use while playing games (18, 19).

However, evidence of the efficacy of this approach remains limited (20, 21). Compliance rates remain low and improvements in binocular vision function remain unsatisfactory. Training video games have limited capacities for customization and their efficacy is affected by users' levels of self-control; alternatives are required. Additionally, the method of rebalancing the eyes' contrast lacks theoretical basis, possibly because the poor eyesight produces fuzzy images that cannot reach the training effect and the artificially low contrast cannot achieve binocular balance. Given these limitations, we proposed an approach based on virtual reality (22–24) and the EYEBIT mobile phone software system. This system is portable, is easy to use, and includes rich content that can be personalized; its use can be monitored remotely by physicians supporting patient training. This approach also enables parents to upload educational videos, which can be used as part of training, supporting children's learning. More importantly, EYEBIT adopts the partial matching method by adjusting the size of the central area of the good eye picture, which allows patients to not only perceive the complete picture but also increase the visual stimulation of the macula center to train and improve the vision and binocular visual function of amblyopia. In the clinical study, we collected 76 patients, 74 of whom had no adverse reaction to the EYEBIT training method. However, two patients experienced fatigue and eye distension during training. After inspection, we found that there was an error in the adjustment of pupil distance. After the VR box was reset to the patient's appropriate pupil distance, the symptoms disappeared.

Compliance is the key factor affecting amblyopia treatment. This study aimed to compare parent and child compliance scores associated with the traditional patching and EYEBIT methods, both of which were experienced by all children in random order. The compliance assessment questionnaire was evaluated to confirm its suitability for factor analysis. Using factor analysis to test validity requires strong correlations among the questionnaire items indicated by the KMO and Bartlett spherical test values. The KMO value compares simple and partial correlation coefficients among the items, yielding values between 0 and 1. Suitability for factor analysis can be high, moderate, low, and none, corresponding to the KMO values of ≥0.9, 0.7–0.9, 0.6–0.7, and <0.6, respectively. The KMO and Bartlett spherical test values were both >0.7, suggesting a strong correlation among items. To further test questionnaire reliability, we examined the internal consistency of the results, using Cronbach's alpha, which revealed that the subjects' answers to the questionnaire items were consistent. Given good reliability and validity of the assessment method, we compared compliance and psychological acceptance of the participating children and their parents, showing differences between the methods used (*p* < 0.05). These findings suggest that the EYEBIT method is associated with better compliance than the traditional patching method.

Finally, to examine associations among compliance components, we used Pearson's product-moment correlation analysis. A Pearson correlation coefficient of <0.4, 0.4–0.7, and >0.7 indicates a low, moderate, and high correlation, respectively. The analysis revealed many correlations between compliance components under the two methods. For example, there were significant medium and low positive correlations between children's compliance behavior and children's compliance attitude as well as parents' compliance behavior, respectively, under the traditional patching method. This indicates that a child's cooperation under the traditional patching method is greatly related to whether the child accepts this method, as well as appropriate parental supervision and behavioral intervention, in the treatment process. Further, if parents are not involved in supervision and intervention, it is difficult for a child to actively cooperate under the traditional patching method. Additionally, there were significant low positive correlations among parents' compliance attitude, children's compliance attitude, and parents' compliance behavior under the traditional patching method. This indicates that, for the traditional patching method, the parents' attitude regarding cooperation with the treatment process affects their supervision and behavioral intervention in the treatment of their child, as well as the child's attitude, rendering it is easier to accept the treatment.

In addition, there was a significant low positive correlation between children's EYEBIT compliance and patching compliance. This suggests that without considering the influence of other attitude factors, children who cooperated with the treatment process under the traditional patching method were better able to cooperate under the EYEBIT method. Furthermore, there was a significant moderate positive correlation between parental EYEBIT attitude and patching attitude, and a significant low positive correlation between parental EYEBIT attitude and childrens' EYEBIT compliance. These results suggest a consistent parental attitude toward the treatment of amblyopia, whether by EYEBIT or patching. In other words, parents who support the traditional patching method can similarly support the EYEBIT method. When parents are very supportive of the EYEBIT method, the child's behavioral cooperation is better and the treatment is more active.

Finally, there were significant negative correlations between children's EYEBIT compliance and parental patching compliance, suggesting that for children with a more accepting attitude under the EYEBIT method, their parents had worse compliance under the traditional patching method. Thus, when a child's attitude to EYEBIT is acceptable, they may be more interested in this method, indirectly reflecting the EYEBIT advantage in terms of compliance. For parents and children who do not cooperate or support the traditional patching method, the EYEBIT method may be more accepted and supported by parents, fostering better behavioral cooperation by the child in the treatment process.

## Conclusions

In summary, this study presented a training method associated with high compliance scores among patients and their parents. The EYEBIT mobile phone software system can be managed remotely by physicians supporting patient training, providing a viable option in times of social distancing, infectious disease outbreak, and pandemic management. Based on the advantages of EYEBIT method in the compliance of children's amblyopia treatment, the effectiveness of this method in the treatment of children's amblyopia will be further studied in the future, especially in the improvement of binocular vision function.

## Data availability statement

The original contributions presented in the study are included in the article/Supplementary material, further inquiries can be directed to the corresponding author/s.

## Ethics statement

The studies involving human participants were reviewed and approved by the Fujian Provincial Hospital Ethics Committee (K2018-12-031). Written informed consent to participate in this study was provided by the participants' legal guardian/next of kin.

## Author contributions

Concept and design: LL and GL. Acquisition and analysis or interpretation of data: TL and YX. Drafting of the manuscript: LL. Statistical analysis: LL and HX. Methodology: GL, LL, YX, and HX. Administrative and technical or material support: All authors. All authors read and approved the final manuscript.

## Funding

The work was supported in part by Startup Fund for scientific research, Fujian Medical University (grant number 2018QH1159) to LL and National Institutes of Health (grant number R43EY028778) to GL.

## Conflict of interest

Author GL: pending patent. Author GL is a co-founder of EyeNexo LLC, a startup company developing smartphone apps for vision tests. The mobile app used in this study was developed previously through a collaboration between Mass Eye and Ear and EyeNexo LLC, funded NIH grant R43EY028778. Author GL's financial interests are reviewed and managed by Mass Eye and Ear and Partners Health Care in accordance with their conflict-of-interest policies. EyeNexo currently does not have any commercial product or license related to the subject matter of this article. Author GL did not participate in data collection. The remaining authors declare that the research was conducted in the absence of any commercial or financial relationships that could be construed as a potential conflict of interest.

## Publisher's note

All claims expressed in this article are solely those of the authors and do not necessarily represent those of their affiliated organizations, or those of the publisher, the editors and the reviewers. Any product that may be evaluated in this article, or claim that may be made by its manufacturer, is not guaranteed or endorsed by the publisher.
