# Supplementary material for: Comparison of compliance among patients with pediatric amblyopia undergoing virtual reality-based and traditional patching method training
Source: Front Public Health. 2022 Oct 14;10:1037412. doi: 10.3389/fpubh.2022.1037412 (PMC9614073; doi:10.3389/fpubh.2022.1037412)
Supplement: Supplementary file 1 [file Table_1.DOC]

Questionnaire on compliance with amblyopia treatment

**Children’s compliance behavior assessment:**

1. Wearing glasses: The child can insist on wearing glasses every day

Strongly disagree ___ Disagree ___ Neutral___

Strongly agree ___ Agree ___

2. Covering: Children can always cover their eyes as required

Strongly disagree ___ Disagree ___ Neutral___

Strongly agree ___ Agree ___

3. EB training: children can complete the training seriously every day

Strongly disagree ___ Disagree ___ Neutral___

Strongly agree ___ Agree ___

4. Children are easily trained in masking when first starting treatment

Strongly disagree ___ Disagree ___ Neutral___

Strongly agree ___ Agree ___

5. Children are easily trained in EB when they first start treatment

Strongly disagree ___ Disagree ___ Neutral___

Strongly agree ___ Agree ___

6. In the first week of treatment, the child can cooperate to complete the tasks assigned by the masking training

Strongly disagree ___ Disagree ___ Neutral___

Strongly agree ___ Agree ___

7. In the first week of treatment, the child can cooperate to complete the tasks assigned by the EB training

Strongly disagree ___ Disagree ___ Neutral___

Strongly agree ___ Agree ___

**Children’s compliance attitude assessment:**

Assessment of compliance attitudes in children:

1. Cognitive: Children think their eyes are sick when they cover

Strongly disagree ___ Disagree ___ Neutral___

Strongly agree ___ Agree ___

2. Cognitive: Children will think their eyes are sick during EYEBIT training

Strongly disagree ___ Disagree ___ Neutral___

Strongly agree ___ Agree ___

3. Children do not need to be forced to cover up training often

Strongly disagree ___ Disagree ___ Neutral___

Strongly agree ___ Agree ___

4. Children do not need to be forced to perform EYEBIT training often

Strongly disagree ___ Disagree ___ Neutral___

Strongly agree ___ Agree ___

5. Habits: Children can accept correct eye habits through covering training

Strongly disagree ___ Disagree ___ Neutral___

Strongly agree ___ Agree ___

6. Habits: Children can receive correct eye habits during EB training

Strongly disagree ___ Disagree ___ Neutral___

Strongly agree ___ Agree ___

**Parents’compliance behavior assessment:**

1. Importance: You always supervise and check that your child completes the masking training

Strongly disagree ___ Disagree ___ Neutral___

Strongly agree ___ Agree ___

2. Importance: You always supervise and check your child to complete EB training

Strongly disagree ___ Disagree ___ Neutral___

Strongly agree ___ Agree ___

3. Stability: You can arrange a relatively fixed time and place for covering training for your child

Strongly disagree ___ Disagree ___ Neutral___

Strongly agree ___ Agree ___

4. Stability: You arrange a relatively fixed EB training time and place for your child

Strongly disagree ___ Disagree ___ Neutral___

Strongly agree ___ Agree ___

5. Ongoing: You and your child are always reviewed as required

Strongly disagree ___ Disagree ___ Neutral___

Strongly agree ___ Agree ___

**Parents’compliance attitude assessment:**

1. Study: You can often communicate with other parents or doctors of children with low vision

Strongly disagree ___ Disagree ___ Neutral___

Strongly agree ___ Agree ___

2. Cognitive: You know what amblyopia is

Strongly disagree ___ Disagree ___ Neutral___

Strongly agree ___ Agree ___

3. Confidence: You think you should stick with your current covering training treatment

Strongly disagree ___ Disagree ___ Neutral___

Strongly agree ___ Agree ___

4. Confidence: You think you should stick to your current EB training treatment

Strongly disagree ___ Disagree ___ Neutral___

Strongly agree ___ Agree ___

5. Cooperate: You can more easily accept mask training

Strongly disagree ___ Disagree ___ Neutral___

Strongly agree ___ Agree ___

6. Cooperation: You can more easily accept EB training

Strongly disagree ___ Disagree ___ Neutral___

Strongly agree ___ Agree ___

7. Cooperate: You are concerned that cover-up training will harm your child

Strongly disagree ___ Disagree ___ Neutral___

Strongly agree ___ Agree ___

8. Cooperate: You are worried that EB training will harm your child

Strongly disagree ___ Disagree ___ Neutral___

Strongly agree ___ Agree ___
